# Altered Immune Activation and IL-23 Signaling in Response to *Candida albicans* in Autoimmune Polyendocrine Syndrome Type 1

**DOI:** 10.3389/fimmu.2017.01074

**Published:** 2017-09-01

**Authors:** Øyvind Bruserud, Eirik Bratland, Alexander Hellesen, Nicolas Delaleu, Håkon Reikvam, Bergithe E. Oftedal, Anette S. B. Wolff

**Affiliations:** ^1^Department of Clinical Science, University of Bergen, Bergen, Norway; ^2^Broegelmann Research Laboratory, Department of Clinical Science, University of Bergen, Bergen, Norway; ^3^Swiss Institute of Bioinformatics, Lausanne, Switzerland; ^4^Computational Biology Unit, Department of Informatics, University of Bergen, Bergen, Norway; ^5^Department of Medicine, Haukeland University Hospital, Bergen, Norway

**Keywords:** autoimmune polyendocrine syndrome type 1, chronic mucocutaneous candidiasis, monocytes, IL-17, IL-22, IL-23

## Abstract

**Objective:**

Autoimmune polyendocrine syndrome type 1 (APS-1) is a rare, childhood onset disease caused by mutations in the *autoimmune regulator* (*AIRE*) gene. Chronic mucocutaneous candidiasis (CMC) is one of the three major disease components and is, to date, mainly explained by the presence of neutralizing auto-antibodies against cytokines [interleukin (IL)-17A, IL-17F, and IL-22] from T helper 17 cells, which are critical for the protection against fungal infections. However, patients without current auto-antibodies also present CMC and we, therefore, hypothesized that other immune mechanisms contribute to CMC in APS-1.

**Methods:**

Whole blood was stimulated with *Candida albicans* (*C. albicans*) in a standardized assay, and immune activation was investigated by analyzing 46 secreted immune mediators. Then, peripheral blood mononuclear cells were stimulated with curdlan, a Dectin-1 agonist and IL-23 inducer, and the IL-23p19 response in monocytes was analyzed by flow cytometry.

**Results:**

We found an altered immune response in APS-1 patients compared with healthy controls. Patients fail to increase the essential ILs, such as IL-2, IL-17A, IL-22, and IL-23, when stimulating whole blood with *C. albicans*. A significantly altered IL-23p19 response was detected in patients’ monocytes upon stimulation with curdlan.

**Conclusion:**

APS-1 patients have an altered immune response to *C. albicans* including a dysregulation of IL-23p19 production in monocytes. This probably contributes to the selective susceptibility to CMC found in the majority of patients.

## Introduction

Autoimmune polyendocrine syndrome type 1 (APS-1) or autoimmune polyendocrinopathy-candidiasis-ectodermal dystrophy (OMIM 240300) is clinically defined by the presence of two of the three major disease components primary adrenal insufficiency, hypoparathyroidism (HP), and chronic mucocutaneous candidiasis (CMC) (1). However, the phenotypic expression of the syndrome is highly variable and includes many minor disease components (2). All patients present auto-antibodies against autoantigens expressed in the affected tissue and/or against immune mediators (3–5). The *autoimmune regulator* (*AIRE)* gene is the disease-causing gene (6–8). AIRE acts as a transcription factor and is almost exclusively expressed in the thymus (9), where it orchestrates the process of negative selection of self-reactive T cells and contributes to the development of regulatory T cells (Tregs) (10, 11).

*Candida albicans* (*C. albicans*) is an opportunistic yeast, colonizing the skin and mucosa of most healthy individuals without causing tissue damage or disease (12). However, it may cause superficial mucocutaneous or systemic infections; often in individuals with impaired immune functions. In APS-1, CMC caused by *C. albicans* is the most common and earliest main manifestation (13) and is reported in 75–100% of patients (1, 4, 14–17). The clinical course varies from periodic to chronic and usually affects the oral mucosa as angular chelitis, or the whole mouth with hypertrophic and/or atrophic lesions (1, 2, 13). Skin, nails, and genital mucosa in females may also be affected. Susceptibility to candidiasis maps to mucosal, not systemic, disease in APS-1 (1).

Interleukin (IL)-23 is required for differentiation, function, and maintenance of T helper 17 (Th17) cells, and this signaling axis plays a central role in host defense against cutaneous candidiasis (18). In APS-1, neutralizing auto-antibodies against the Th17 cytokines, IL-17A, IL-17F, and IL-22, are suggested to explain the impairment in mucosal immunity (19, 20). Noteworthy, patients without auto-antibodies also present CMC (4), and therefore, it remains disputable whether the Th17 cytokine-neutralizing auto-antibodies are sufficient to precipitate CMC. To gain a better understanding of the molecular mechanism of CMC in APS-1, we investigated the immune activation in response to *C. albicans* in both whole blood and monocytes of patients finding a generally altered immune activation including a dysregulation in the IL-23/Th17 pathway.

## Materials and Methods

### Patients and Clinical Data

Patients (*n* = 18) were included from our National Registry of Organ Specific Autoimmune Diseases and were previously described in the Norwegian cohort (4, 21, 22). All fulfilled the diagnostic criteria of APS-1 (2). Patients received appropriate hormone replacement therapy of endocrine deficiencies at physiological doses. In HP, normal levels of calcium were maintained with oral administration of cholecalciferol and calcium. However, these treatments should not have significant immunomodulatory effects. None of the patients or healthy controls was pregnant, had acute infections, or received vaccinations at the time of sampling. An overview of the patients’ data is given in Table 1. Samples from all patients were not available for all experiments. Healthy age and gender matched controls (*n* = 31) were recruited from the local blood bank at Haukeland University Hospital. All participants gave informed and written consent, and the study was approved by The Regional Committee for Medical and Health Research Ethics for Western Norway.

**Table 1:** Characterization of the autoimmune polyendocrine syndrome type 1 (APS-1) patients.

Patient number	Family number	Sex	Year of birth (YoB)	Age of onset	Classic triad	Other manifestations	Autoimmune regulator (AIRE) mutations	IFNω auto-antibodies	Other auto-antibodies
1	I	M	1995	3	CMC(3), HP(4), PAI(12)	Al(4), TIN(15), AT(16), E	C.967_979del13/c.769C>T	Positive	21OH, IL-17, IL-22, TGM4
2	I	M	1992	2	CMC(2), HP(4)	K(11), M(15), E	C.967_979del13/c.769C>T	Positive	AADC, GAD65, IL-17, IL-22, TGM4, TH
3	II	F	1958	5	CMC(5), HP(9), PAI(14)	G(18), AS(43), TIN(47), E(53)	C.967_979del13/large del	Positive	21OH, 17OH, IL-22, MAGEB2, NALP5, SCC, TH
4	II	F	2002	7	PAI(7), HP(10), CMC	E, M	C.967_979del13/c.967_979del13	Positive	21OH, 17OH, AADC, GAD65, IL-22, MAGEB2, NALP5, SCC, TH, TPH1
5	III	M	1948	7	CMC(7), HP(9), PAI(16)	V(17), Al(21), B12(63), E	C.769C>T/c.769C>T	Positive	21OH, AADC, IL-17, IL-22 MAGEB2, SCC, SOX10, TGM4
6	IV	F	1960	9	HP(9), CMC	Al(6), G(17), AT, E, N	C.22C>T/c.290T>C	Positive	NALP5, PCA
7	V	M	1970	12	PAI(12), CMC(42)	E	C.967_979del13/c.967_979del13	Positive	21OH, GAD65, IL-22, SCC
8	VI	F	1974	23	PAI(23), CMC(23)	E	C.879+1G>A/c.879+1G>A	Positive	21OH, 17OH, NALP5
9	VI	M	1959	43	HP(43), CMC	V(15), DM(32), E(49), AT(51)	C.879+1G>A/c.879+1G>A	Positive	21OH, 17OH, AADC, GAD65, NALP5, TH, TPH1
10	VII	M	1964	14	HP(14), CMC(22)	DM(23), K(25), N(25), V(41), Al(41), E	C.769C>T/c.1249dupC	Positive	AADC, GAD65, IL-22, PCA, PDILT, TGM4, TH, TPH1
11	VII	M	1963	nk	CMC?	E	C.769C>T/c.1249dupC	Positive	AADC, IL-22, SOX10, TGM4
12	VIII	F	1988	3	HP(3)	AT(24), E, M	C.967_979del13/c.967_979del13	Positive	NALP5
13	IX	F	1987	2	CMC(2), HP(15)	E(24), Al, E	C.1163_1164insA/c.1249_1950dupC	Positive	21OH, AADC, IL-17, IL-22, MAGEB2, NALP5, SOX10
14	X	F	1971	5	HP(5)	G(19), B12(35), M(39), E	C.934G>A/not found	Positive	NALP5, AADC, GAD, PCA
15	XI	F	1976	4	HP(4), C	E(14), AT(20), V(25)	C.967-979del13/c.967-979del13	Positive	21OH, 17OH, NALP5, TH, TPH, AADC, GAD, SCC, MAGEB2, SOX10, PDILT, IL-22
16	XI	M	1980	9	HP(9), PAI(12), C(16)	E	C.967-979del13/c.967-979del13	Positive	21OH, SCC, TH, AADC, GAD, NALP5, TGM4, IL-17, IL-22
17	XII	M	1958	Not known	PAI(55), HP, C	Al, AS, E	C.967-979del13/c.967-979del13	Positive	GAD, TPH, MAGEB2, IL-17, IL-22
18	XIII	F	1982	5	CMC(3)	V(15), PA(13), E, M	C.967-976del13/c.977C>T	Positive	AADC, GAD65, IL-22, PCA, PDILT, TPH1

*Patient number, family, sex (M, male; F, female), YoB, clinical manifestations, *AIRE* mutations and auto-antibodies in APS-1 patients. The age of debut denotes the age at which the first APS-1 main component appeared. The age at diagnosis is written in parentheses*.

*21OH, 21-hydroxylase; 17OH, 17-α-hydroxylase; AADC, aromatic l-amino acid decarboxylase; Al, alopecia; AS, asplenism; AT, autoimmune thyroiditis; CMC, candidiasis; DM, diabetes mellitus; E, enamel hypoplasia; G, hypogonadism; GAD65, glutamic acid decarboxylase 65-kDA isoform; HP, hypoparathyroidism; IFNω, interferon-omega; IL-17, interleukin-17; IL-22, interleukin-22; K, keratoconjunctivitis; M, malabsorption; MAGEB2, melanoma antigen B2; N, nail hypotrophy; NALP5, NACHT leucine-rich-repeat protein 5; PA, pernicious anemia; PAI, primary adrenocortical insuffiency; PCA, parietal cell antigen; PDILT, protein disulfide isomerase-like testis expressed; SCC, side-cleavage enzyme; SOX10, sex-determining region Y-box 10; TGM4, transglutaminase 4; TIN, nephritis; TH, tyrosine hydroxylase; TPH, tryptophan hydroxylase; V, vitiligo*.

### Measurement of Immune Mediators

*In vitro* production of immune mediators in response to *C. albicans* was characterized using the TrueCulture assay system (Myriad, RBM, USA). One milliliter of whole blood was taken from APS-1 patients (*n* = 11) and age- and sex-matched healthy controls (*n* = 13) into TrueCulture collection and culture tubes (Myriad, RBM) that contained either the supplied media (baseline response) or media supplemented with *C. albicans* (ATCC 10231; Myriad, RBM). Following a 48 h activation period at 37°C, the supernatants were frozen and sent to Myriad RBM’s testing laboratory where 46 unique analytes or biomarkers of immune activation were assessed using the multiplex immunoassay Human InflammationMAP 1.0 panel (Myriad, RBM). Mediators of special interests (IL-17A, IL-17F, IL-22, and IL-23p19) were also assayed with enzyme-linked immunosorbent assay (ELISA) (R&D Systems, UK) for all patients included in this experiment (*n* = 11) and an extended group of healthy controls (*n* = 19). These ELISAs were done on supernatants from the corresponding TrueCulture tube. Standard sandwich ELISA was performed on sera from all patients (*n* = 18) searching for auto-antibodies against IL-23 (PeproTech, USA).

### Isolation and Culture of Cells

Peripheral blood mononuclear cells from APS-1 patients (*n* = 6) and healthy controls (*n* = 12) were isolated from heparinized blood by Ficoll-Paque PLUS (GE Healthcare) density gradient centrifugation and stored at −150°C. Cryopreserved peripheral blood mononuclear cells (PBMCs) were used in all cell experiments. PBMCs (10^6^ cells/mL) were cultured overnight in RPMI-1640 medium (Lonza) supplemented with 10% human AB serum (Sigma, USA) and 1% penicillin–streptomycin (Sigma) at 37°C with 5% CO_2_. For the immune stimulation and activation of monocytes, 10 μg/mL of the Dectin-1 agonist beta-1,3-glucan (Curdlan AL, InvivoGen, USA) was added to the cell cultures. Lipopolysaccharide from *Salmonella abortus equi* (Sigma-Aldrich) in a concentration of 1 μg/mL was used as a non-*Candida* positive control for monocyte activation. The protein transport inhibitor Brefeldin A (BioLegend, USA) was added at 1 μg/mL 90–120 min after the start of incubation.

### Flow Cytometry

After the incubation period, cells were washed with 2 mL buffer [phosphate-buffered saline (Sigma) containing 5% fetal bovine serum (Life technologies)] and centrifuged at 350 × *g* for 5 min. Cell surface staining of monocytes was done in 0.1 mL buffer for 30 min on ice with fluorescein isothiocyanate-conjugated antibody to human CD14 (clone HCD14, BioLegend) at a dilution of 1:20. Cells were then washed and fixed in 0.5 mL/tube Fixation Buffer (BioLegend) in the dark for 20 min at room temperature before centrifugation at 350 × *g* for 5 min and then washed again. The fixed cells were re-suspended in 0.1 mL Intracellular Staining Perm Wash Buffer (BioLegend) and stained with 10 μL phycoerythrin-conjugated antibody to human IL-23p19 (clone #727753; R&D Systems) in the dark for 20 min at room temperature. Finally, another washing step was done before the fixed and stained cells were re-suspended in 0.3 mL buffer. Cells were analyzed on a BD FACS Accuri C6 flow cytometer. Individual populations were gated according to forward scatter (FSC), side scatter (SSC), and specific markers, and the data were subsequently analyzed with FlowJo X software.

### Statistical and Bioinformatical Analyses

Paired *t*-tests were used analyzing paired data, and the Mann–Whitney *U* test was used when comparing groups. The level of significance was defined to a *P* value less than 0.05. Statistical analyses were performed using IBM SPSS Statistics 23 or Prism 7 (Graph Pad Software, Inc., San Diego, CA, USA). Hierarchical cluster analyses were performed using the J-Express (MolMine AS, Bergen, Norway) (23), and the alterations analyzed were standardized after ratio after/before stimulation and log(2) transformed before unsupervised hierarchical clustering with Squared Euclidean distance measure with weighted average linkage was performed (23). Functional annotation and generation of the basic framework of the network displayed in Figure S1 in Supplementary Material were computed using ClueGO 2.3.3 (24) within the Cytoscape 3.4.0 suite (25). Proteins that in APS-1 patients failed to be up-regulated in response to *C. albicans* (*P* > 0.05) while found to be significantly increased in healthy controls upon *C. albicans* exposure (*P* < 0.05) were entered as group 1. Group 2 includes SERPINA1 and GC, which were found significantly down-regulated in APS-1 patients but were unchanged in healthy subjects when responding to *C. albicans* stimulation. These two groups were interrogated regarding their associations with *a priori*-defined biological terms comprised in the complete biological process gene ontology (GO) (GO:0008150; 23.02.2017). The analysis settings were (i) hits required to map a GO term ≥1, (ii) percentage of a term covered by proteins reliably detected by the MAPs ≥4%, and (iii) GO term fusion and GO term grouping was applied based on a kappa score of 0.4 (similarity measure between the GO terms). For each group of terms, the GO term accommodating the largest number of proteins was selected as its leading term. All other settings were used as per default.

## Results

In the TrueCulture assay, a total of 46 single mediators were analyzed in supernatants after the activation period of 48 h. Table 2 gives an overview of all mediators analyzed, the response to *C. albicans* stimuli in patients and healthy controls, and *P* values for paired analyses within each group.

**Table 2:** Change of different immune mediators in response to *Candida albicans* in patients and controls.

Mediator	Autoimmune polyendocrine syndrome type 1		Controls	
	Response to *C. albicans*	*P* value	Response to *C. albicans*	*P* value
**Interleukins**				
IL-1A	↑	0.001	↑	0.001
IL-1B	↑	0.001	↑	<0.001
**IL-2**	**→**	**Ns (0.115)**	**↑**	**0.001**
IL-3	Nd	–	Nd	–
IL-4	↑	<0.001	↑	<0.001
IL-5	Nd	–	Nd	–
IL-6	↑	0.014	↑	0.002
IL-7	↑	0.001	↑	0.037
IL-10	↑	0.008	↑	0.003
IL-12p40	↑	0.020	↑	<0.001
IL-12p70	Nd	–	Nd	–
IL-15	Nd	–	Nd	–
**IL-17A**	**→**	**Ns (0.115)**	**↑**	<**0.001**
IL-18	↑	<0.001	↑	<0.001
**IL-22^a^**	**→**	**Ns (0.240)**	**↑**	**0.002**
**IL-23**	**→**	**Ns (0.342)**	**↑**	**0.035**
**Growth factors**				
BDNF	→	Ns (0.463)	→	Ns (0.203)
CSF2	↑	0.007	↑	0.002
VEGFA	↑	0.001	↑	<0.001
**Chemokines**				
CCL2	↑	0.002	↑	<0.001
CCL3	↑	0.001	↑	<0.001
CCL4	↑	<0.001	↑	<0.001
CCL5	→	Ns (0.514)	→	Ns (0.072)
CCL11	↓	0.014	↓	0.004
CXCL8	↑	<0.001	↑	<0.001
**Immunomodulatory cytokines**				
INFg	↑	0.003	↑	0.014
LTA	↑	<0.001	↑	<0.001
TNF	↑	0.002	↑	<0.001
**Adhesion molecules**				
ICAM1	→	Ns (0.582)	→	Ns (0.377)
VCAM1	→	Ns (0.401)	→	Ns (0.546)
**Matrix metalloproteases**				
MMP3	→	Ns (0.740)	→	Ns (0.247)
MMP9	↑	0.003	↑	0.004
**Coagulation factors**				
**C3**	**→**	**Ns (0.661)**	**↑**	**0.030**
F7	→	Ns (0.060)	→	Ns (0.101)
**FGA**	**→**	**Ns (0.450)**	**↑**	**0.011**
vWF	→	Ns (0.807)	→	Ns (0.464)
**Serum proteins**				
B2M	**→**	**Ns (0.637)**	**↑**	**0.024**
FTL	↑	0.002	↑	<0.001
HP	→	Ns (0.562)	→	Ns (0.967)
**Traditional markers**				
CRP	→	0.124	→	0.725
**Others**				
**GC**	↓	**0.037**	**→**	**Ns (0.974)**
**SERPINA1**	↓	**0.028**	**→**	**Ns (0.683)**
IL1RN	↑	<0.001	↑	<0.001
KITL	↑	0.002	↑	<0.001
TIMP1	↑	0.017	↑	<0.001
TNFRSF1B	↑	<0.001	↑	<0.001

*The table gives the mediators analyzed in the TrueCulture system and the response to C. albicans in both patients and healthy controls. T-test for paired data was used for statistical comparison. ↑ indicates a significant up-regulation, ↓ indicates a significant down-regulation, and → indicates no significant change in the mediator concentration. Mediators with a significant different response in patients and controls are marked bold*.

*Nd, not detectable; Ns, not significant*.

*^a^IL-22 was not included in the TrueCulture system but analyzed with enzyme-linked immunosorbent assay*.

### Most Chemokines, Interleukins, and Immune Regulatory Cytokines Increase in Response to *C. albicans* in Both Patients and Healthy Controls

**Figure 1:**
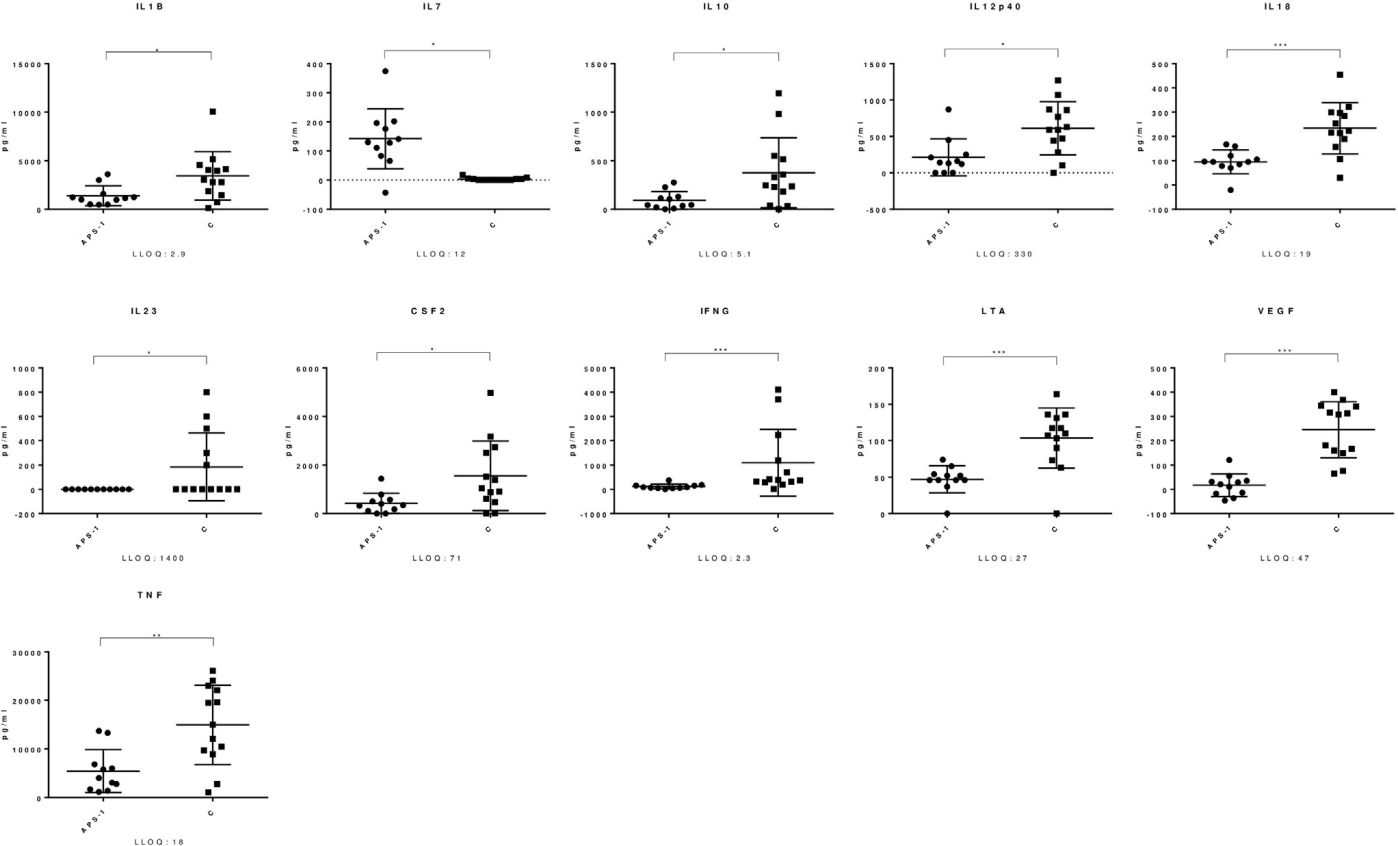
Mediators with significant different response in Autoimmune polyendocrine syndrome type 1 (APS-1) and controls. The figure shows selected mediators from the TrueCulture assay and significant differences were found comparing APS-1 and controls (C). The lines indicate the mean with standard deviation. Concentrations are given at the *y*-axis. LLOQ, lower limit of quantitation. *P ≤ 0.05; **P ≤ 0.01; ***P ≤ 0.001.

Both APS-1 patients and healthy controls had a significant increase in levels of several mediators in response to *C. albicans*. A significant up-regulation in APS-1 and controls was found in the analyzed γ-chemokines CCL2, CCL3, and CCL4, the α-chemokine CXCL8, and the interleukins IL-1β, IL-4, IL-6, IL-7, IL-10, IL-12p40, and IL-18 (Table 2). Similarly, IL-1RN, the three immune regulatory cytokines LTA, TNF, and IFNγ and the growth factors CSF2 and VEGF were found to be significantly increased in both groups. Notably, when comparing the responses in the two groups, significant differences were found for 16 mediators (Table S1 in Supplementary Material; Figure 1). CCL11 was the only mediator analyzed, which significantly decreased in response to *C. albicans* in both groups. Finally, a total of 11 mediators analyzed remained unchanged in both groups after stimulation of whole blood by *C. albicans* (Table 2).

### APS-1 Patients Have an Altered Th17 Cytokine Response to *C. albicans* Compared to Healthy Controls

A subset of related mediators failed to increase in response to *C. albicans* in APS-1 patients compared to healthy controls. Interestingly, patients fail to increase the essential interleukins IL-17A, IL-22, and IL-23 (Table 2). IL-22 was not included in the TrueCulture assay and, therefore, analyzed with standard ELISA on the TrueCulture supernatants, and IL-17A and IL-23 were analyzed in both assays with comparable results. In controls, the strength of IL-22 correlated with IL-17A (correlation 0.869, *P* = 0.001) and IL-23 correlated with IL-17F (correlation 0.612, *P* = 0.026). These correlations could not be observed in patients. No APS-1 patients or controls had auto-antibodies against IL-23 assayed by sandwich ELISA. The other mediators revealing a different response in patients were β2-microglobulin (B2M), C3, and FGA, whereas GC and SERPINA1 remained unchanged in healthy controls and decreased significantly in APS-1 patients. All mediators with a significant different response comparing patients and controls are highlighted in Table 2.

### Unsupervised Hierarchical Clustering Based on Changes in Immune Mediators and Functional Annotation of the Protein Profile Characterizing the Altered Immune Response in APS-1 Patients

**Figure 2:**
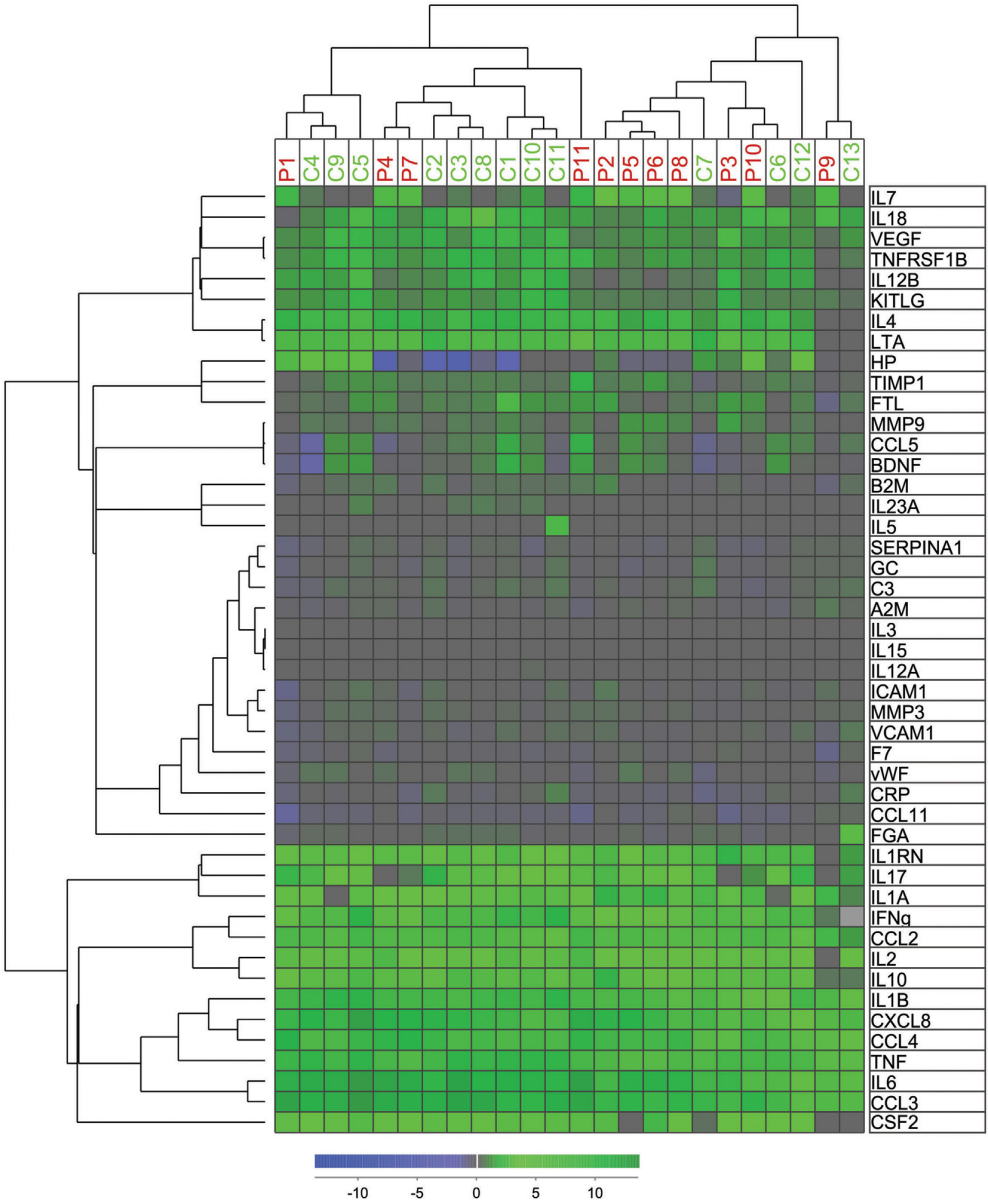
Hierarchical cluster analyses of mediator alteration in control and patients. Unsupervised hierarchical cluster analyses and distance matrix analyses for 11 patients (red P1–P11) and 13 controls (green C1–C13) were performed. Concentrations of mediators were measured before and after intervention as previously described. The alterations were standardized after ratio after/before intervention and log(2) transformed before unsupervised hierarchical clustering with Squared Euclidean distance measure with weighted average linkage was performed, resulting in a heat map for visualization and interpretation. The mediator alteration profiles identified to main patients/control clusters and two main cytokine clusters.

We used the relative values of the measured levels of all mediators analyzed in the TrueCulture assay in an unsupervised hierarchical clustering analysis (Figure 2). Patients and controls were divided into two main clusters/subsets, and most patients were included in the left cluster (7 of 11) and most controls in the right cluster (9 of 13). However, no significant difference was found when comparing expected and observed frequencies using the Chi-square test (*P* = 0.107). Figure S1 in Supplementary Material shows a protein:gene ontology-(GO) term network, which identified four clusters of biological processes, each with its leading term, that most efficiently interconnect the nine proteins showing significantly different responses to *C. albicans* among patients and controls. Any regulatory relationships between the proteins, as per CluePedia v10.0, are displayed as well.

### Monocytes from APS-1 Patients Have an Impaired IL-23p19 Response When Stimulated with Curdlan

**Figure 3:**
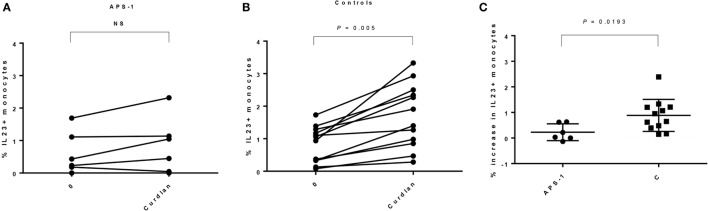
Interleukin (IL)-23p19 responses found in monocytes in autoimmune polyendocrine syndrome type 1 (APS-1) patients and controls. The figure gives an overview of the IL-23p19 response found in monocytes in patients versus controls. **(A)** APS-1 patients; no significant increase in IL-23p19 was detected in monocytes. Numbers at the *Y*-axis gives the percentages of IL-23p19^+^ monocytes after stimulation with curdlan. **(B)** Controls; a significant increase in IL-23p19 was detected in monocytes. The numbers at the *Y*-axis give the percentages of IL-23p19^+^ monocytes after stimulation with curdlan. **(C)** Comparing the increase in IL-23p19^+^ monocytes found in APS-1 and controls at baseline and after curdlan stimulation. NS, not significant.

We found comparable numbers of monocytes in unstimulated PBMCs from patients and controls (Table 3), and no significant difference in frequencies of IL-23p19^+^ cells was found comparing unstimulated cultures. Although not significant, patients seem to have a greater variability in both numbers of monocytes when gating on FSC/SSC and CD14^+^ monocytes (Table 3; Figure S2 in Supplementary Material). However, when comparing unstimulated and stimulated monocytes, the levels of IL-23p19 were significantly increasing in controls, whereas this was not found in APS-1 (Figures 3A,B; Figure S2 in Supplementary Material). Moreover, healthy controls had a significant greater increase in total IL-23p19^+^ monocytes upon stimulation with curdlan (Figure 3C).

**Table 3:** Monocyte counts in autoimmune polyendocrine syndrome type 1 (APS-1) and controls.

Cells	APS-1	Controls	*P* value
Monocytes [forward scatter (FSC)/side scatter (SCC)]	8.810 (1.98–16.6)	11.70 (8.32–16.00)	Ns (0.0978)
CD14^+^ monocytes	64.9 (29.0–86.90)	79.45 (67.10–87.80)	Ns (0.3355)
Interleukin (IL)-23p19^+^ monocytes – 0	0.33 (0.00–1.69)	1.00 (0.078–1.73)	Ns (0.4796)
IL-23p19^+^ monocytes – curdlan	0.75 (0.00–2.32)	1.655 (0.28–3.33)	Ns (0.0831)

*The table gives a comparison of percent monocytes in APS-1 and controls both when gated on FSC/SSC and the number of CD14^+^ monocytes in the current monocyte gate. Numbers are given as median (and range). The Mann–Whitney *U* test was used for the statistical comparison*.

*0, baseline/unstimulated. Ns, not significant*.

## Discussion

Chronic mucocutaneous candidiasis as a major clinical disease manifestation in APS-1 suggests immune defects in mechanisms crucial in fungal defense. The aims of the present study were to characterize APS-1 patients’ immune activation in response to *C. albicans* and our findings clearly state that APS-1 patients have an altered immune activation compared to healthy controls. IL-23p19, which is required for the formation of Th17 cells, was found dysregulated both in whole blood and monocytes upon stimulation with *C. albicans* indicating its contribution to CMC in patients.

The overall differences detected in the immune mediators in APS-1 patients have relatively broad implications on the quality of the immune response against *C. albicans* (Figure S1 in Supplementary Material). Importantly, with regard to immune responses against *Candida*, biological processes involving positive regulation of IL-17- and IL-23 production, as well as positive regulation of lymphocyte mediated immunity, were clearly impaired in patients. The significance of the other enriched biological processes impaired in patients, positive regulation of T-cell-mediated cytotoxicity, and immunoglobulin-mediated immune responses, respectively, is less obvious. However, a severely impaired general CD8^+^ T-cell homeostasis has been previously reported in APS-1 patients (26). The importance of humoral immunity against *Candida* is suggested to be relatively modest compared to cellular defense mechanisms (27). Still, the most important mediator in the biological process involving immunoglobulin mediated immune responses was the complement factor C3, which APS-1 patients failed to up-regulate in response to *C. albicans*. C3 plays several important roles during the early innate responses against *Candida*, through opsonization and subsequently recognition and phagocytosis by neutrophils and monocytes (28). Moreover, comparing the increase in immune mediators in response to *C. albicans*, significant differences between patients and controls were found for several mediators (IL-1β, IL-10, IL-12p40, IL-18, and IL-23) involved in the crosstalk between innate immune cells and subsets of T cells. This indicates that several cellular mechanisms may be involved in altering the immune response in patients. NOD-like receptors are cytosolic proteins that are implicated in sensing fungi and, once activated, produce IL-1β and IL-18 through the formation of inflammasomes (29), both of which were less up-regulated in patients compared to controls in response to *Candida* stimulation. IL-1β is crucial for the differentiation of Th17 cells and for the activation and recruitment of neutrophil granulocytes, while IL-18 is important for the induction of IFNγ producing T helper 1 (Th1) cells. IL-10 is produced by almost all immune cells and its major role is to limit the extent of immune activation and retain homeostasis (30). IL-12 is a pro-inflammatory molecule primarily produced by professional antigen-presenting cells (31) and it activates natural killer cells and induces the differentiation of naïve CD4+ T lymphocytes to become Th1 cells (31). Notably, the IL-12p40 chain of IL-12 can also form a dimer with p19 giving rise to IL-23 (32), which is required for Th17 differentiation, function and maintenance. Patients failed to increase IL-17A, IL-22, and IL-23, which are found critical for optimal host defense against cutaneous candidiasis (18) and fungal infections (19, 20). Previous studies have reported conflicting results about IL-17 production in APS-1 (20, 33), but the finding of reduced IL-22 producing cells in APS-1 seems more consistent (20, 33, 34). Finally, we also confirm a dysregulation of IL-7, which is previously described in a Finnish APS-1 cohort (26). Although a more comprehensive study of the system biology would generate an even better overview of the immune activation and biological pathways involved in the response to *Candida* in APS-1, our findings indicate an altered immune activation in APS-1 patients, which includes several immune mediators that play important roles in immune homeostasis and particularly in the host defense against fungi (Figure S1 in Supplementary Material).

Interleukin-23 and Th17 cytokines have been implicated in the pathogenesis of many autoimmune diseases (35–37), and we therefore found the total lack of IL-23 in APS-1 patients particularly interesting. IL-23 is secreted by activated monocytes and dendritic cells (38) and induces the differentiation of naïve T cells into Th17 cells and can promote the further expansion and maintenance of Th17 cells, and the production of Th17 cytokines via IL-23 receptor and STAT3 (38). In the literature, there is already conflicting information about IL-23 signaling in APS-1. Ryan and collaborators described monocyte-derived DCs from APS-1 patients to over-produce IL-2, INFγ, TNF-α, and IL-13 and demonstrated both impairment in maturation and hyper activation in response to *C. albicans* (39). However, the IL-23 response was comparable to controls (39). Another study assessed Th17 responses of PBMCs to *Candida* and non-*Candida* species stimuli finding that PBMCs from APS-1 patients had a normal or increased IL-17 production and Th17 cell proliferation, although only in the absence of their own plasma, which had an inhibitory effect on both IL-17 production and Th17 cell proliferation (34). This study reported normal IL-6 and IL-23 responses in APS-1 patients. Furthermore, expression levels of all pattern recognition receptors (PRRs) involved in anti-candida responses, levels of plasmacytoid and myeloid DCs, and monocyte toll-like receptor (TLR)-2/TLR-6 expression are described similar in APS-1 and controls (40). Finally, we have previously reported reduced numbers of CCR6^+^CXCR3^+^ T helper cells, CD16^+^ monocytes, and Tregs in patients with APS-1 (41). These previous studies have investigated IL-23 from DCs and PBMCs in APS-1 (34, 39, 40), but less information about monocytes exists. We report comparable numbers of monocytes in both patients and controls and similar levels of IL-23p19 in unstimulated monocytes. Interestingly, monocytes from patients fail to increase IL-23p19 production both when comparing the steady-state levels and stimulated levels within each group and when comparing the total increase between the groups. The reason for the discrepancy in IL-23 production in the studies mention above and our current findings could be due to differences in cell types studied (PBMCs, DCs, monocytes, and whole blood), or that PRRs activate differently to particular *Candida* strains. These important aspects need to be further investigated.

Furthermore, there are conflicting information about the clinical relevance of monocyte-specific IL-23 signaling and CMC. Patients with autosomal recessive IL-12RB1 or IL-12p40 deficiency suffer from CMC and, therefore, indicate that impairment of IL-23 signaling can be the molecular pathogenesis of CMC (42, 43). On the other hand, CMC is not well described in patients with GATA2 deficiency, which severely impairs monocytes (44). Our findings suggest a monocyte-specific IL-23 deficiency in APS-1 patients, which resonates well with a previously published study reporting an extrathymic role of AIRE in monocytes (45). Specifically, that study described how AIRE interacts with CARD9, SYK, and Dectin-1 in healthy monocytes and thus plays a major role in activation of the Dectin-1 pathway by stimulation with curdlan. Consequently, PBMCs from AIRE-deficient APS-1 patients produced significantly less TNF in response to curdlan stimulation compared to healthy controls. Noteworthy, the expression and biological role of the AIRE protein in peripheral blood cells are controversial (46, 47), and the interpretation of our data in this context should be approached with caution until the expression of AIRE in monocytes has been further verified. Based on the above, a general impairment in immune activation and an altered monocyte response probably contribute to CMC found in APS-1 and is likely to involve the Dectin-1 pathway.

Auto-antibodies play a key role in CMC in APS-1, and auto-antibodies against IL-6, IL-17A, IL-17F, IL-22, and IFNω are previously described (19, 20, 48). We searched for auto-antibodies against IL-23 in all patients without any positive findings, confirming a previous report (48). All patients included in our study present auto-antibodies against INFω, and about 60% have auto-antibodies against IL-22. To speculate, these auto-antibodies may influence the difference in immune response found in whole blood by interacting in autocrine and paracrine signaling loops and thereby disturb cellular responses. These speculations are supported by the finding that APS-1 patients failed to up-regulate the interferon regulated serum inflammation marker B2M after *C. albicans* stimulation. The inhibition of interferon regulated genes by APS-1 patient auto-antibodies is well established (49), and the importance of type I interferons in immunity against *Candida* was recently demonstrated (50).

Our study has some limitations. First, the rarity of APS-1 makes biological samples limited. We therefore had to strictly prioritize our samples and chose to focus on stimulation assays in the search of differences in the protein expression using flow cytometry, rather than for example detecting RNA transcripts using quantitative polymerase chain reactions. Our cell assays were also more robust compared to pilot experiments of culturing PBMCs or whole blood with *C. albicans*, and analyzing supernatants for signaling molecules using ELISA. Second, in our cell assay, few monocytes produced IL-23p19 when stimulated with curdlan and some cells were border line positive for IL-23p19 after stimulation. However, we carefully optimized our assay regarding incubation time, concentration of Dectin, and Brefeldin A stimulation. Moreover, samples from two patients and two controls were always stimulated and analyzed together, we used a consistent gating strategy when analyzing data, and our findings regarding IL-23 were comparable in both the cell assay and the TrueCulture system. In general, whole blood assays probably mirror the *in vivo* conditions of inflammation more precisely than PBMC assays regarding interplay between subsets of immune cells and mediators. We found significantly altered IL-23p19/IL-23 signaling in both our assays making the findings reliable.

In summary, in order to gain further insights into the molecular mechanisms of CMC in APS-1, we used different approaches to investigate the immune activation in APS-1 in response to *C. albicans*. Our findings indicate that patients have a significant altered immune activation with broad implications on the quality of the immune response to *C. albicans* and that monocytes contribute to a dysregulation of the IL-23/Th17 axis, which is crucial for proper immunity to fungal infections.

## Ethics Statement

This study was carried out in accordance with the recommendations of The Regional Committee for Medical and Health Research Ethics with written informed consent from all subjects. All subjects gave written informed consent in accordance with the Declaration of Helsinki. The protocol was approved by The Regional Committee for Medical and Health Research Ethics.

## Author Contributions

ØB, EB, AW, and BO collected samples and clinical information. ØB, HR, and ND did the statistical and bioinformatical analyses. ØB, EB, and AH executed the cell experiments. All authors contributed in writing the manuscript and have approved the final version.

## Conflict of Interest Statement

The authors declare that the research was conducted in the absence of any commercial or financial relationships that could be construed as a potential conflict of interest.

## Supplementary Material

Figure S1 Functional annotation of the protein profile characterizing the altered immune response to *C. albicans* in APS-1 patients versus healthy individuals. This protein:gene-ontology-(GO) term network identified four clusters of biological processes, each with its leading term, that most efficiently interconnect the nine proteins here relevant. Any regulatory relationships between the proteins, as per CluePedia v10.0 are displayed as well.

Figure S2 Gating strategy for flow cytometric analyses.
