# Supplementary material for: Altered Immune Activation and IL-23 Signaling in Response to Candida albicans in Autoimmune Polyendocrine Syndrome Type 1
Source: Front Immunol. 2017 Sep 1;8:1074. doi: 10.3389/fimmu.2017.01074 (PMC5585148; doi:10.3389/fimmu.2017.01074)
Supplement: Supplementary file 1 [file table_1.pdf]

**Supplementary Table 1. Change in immune mediators in response to *C. Albicans* in APS-1 patients and controls.**

The table gives the changes in levels of immune mediators in patients and controls after stimulation of full blood with *Candida albicans*. The table gives the median (and ranges) of the change in mediator levels. The Mann Whitney U test was used for the statistical comparison. Significant differences are marked bold. Lower limit of quantification gives the lowest concentration of an analyte in a sample that can be reliably detected and at which the total error meets the laboratory`s requirements for accuracy. MRBM`s requirement for accuracy is the concentration of an analyte at which the coefficient of variation of replicate standard sample is 30%. All mediator concentrations are given in pg/ml. Nd; not detectable.

| Mediator | Lower limit of quantitation | APS-1 | Controls | P value |
|---|---|---|---|---|
| *INTERLEUKINS* | | | | |
| IL1A | 3.9 | 30 (10-130) | 100 (0.00-410) | 0.2324 |
| **IL1B** | 2.9 | 1139 (472.10-3604) | 3030 (114.90-10050) | **0.0129** |
| IL2 | 8.9 | 156 (0.00-3218) | 175.1 (88.1-827.10) | 0.9429 |
| IL3 | 16 | Nd | Nd | - |
| IL4 | 28 | 52 (0.00-76.00) | 76 (0.00-106) | 0.1627 |
| IL5 | 10 | Nd | Nd | - |
| IL6 | 7.2 | 10796 (389.20-62234) | 24293 (361.8-96285) | 0.1627 |
| **IL7** | 12 | 130 (-43-374) | 0.0 (0.00-18) | **0.0003** |
| **IL10** | 5.1 | 44.20 (0.8-275.2) | 245.9 (1.8-1195) | **0.0154** |
| IL12p70 | 43 | Nd | Nd | - |
| **IL12p40** | 330 | 140 (0.00-870) | 590 (0.00-1270) | **0.0101** |
| IL15 | 720 | Nd | Nd | - |
| IL17 | 3.3 | 8.7 (0.0-153.7) | 22.7 (4.6-76.7) | 0.1224 |
| **IL18** | 19 | 95 (-21-167) | 223 (29-454) | **0.0005** |
| IL22 | - | 67.95 (-226.5-315.0) | 220.7 (-168.8-1287) | 0.1688 |
| **IL23** | 1400 | 0.00 | 0.00 (0-800) | **0.0411** |
| *GROWTH FACTORS* | | | | |
| BDGF | 56 | 0.00 (-2700-3900) | 530 (-5200-6500) | 0.4156 |
| **CSF2** | 71 | 347 (0-1439) | 1049 (0-4969) | **0.0128** |
| **VEGFA** | 47 | 20.9 (-46.3-120.5) | 308.8 (65-400) | **<0.001** |
| *CHEMOKINES* | | | | |
| CCL2 | 24 | 63577 (1784-204535) | 62690 (1058-207566) | 0.8646 |
| CCL3 | 15 | 53464 (3056-162976) | 98769 (2240-225965) | 0.2284 |
| CCL4 | 37 | 177577 (7799-418540) | 263443 (5561-441411) | 0.2066 |
| CCL5 | 52 | -100 (-8000-17300) | 2700 (-14000-28000) | 0.1543 |
| CCL11 | 71 | -44.0 (-210-9) | -21 (-90-29) | 0.4678 |
| CXCL8 | 2.6 | 495742 (11776-951722) | 677380 (25072-1329000) | 0.2518 |
| *IMMUNOMODULATORY CYTOKINES* | | | | |
| **INFG** | 2.3 | 86.40 (1.2-370) | 388.7 (10.7-4108) | **0.0005** |
| **LTA** | 27 | 46 (0-74) | 110 (0-164) | **0.0003** |
| **TNF** | 18 | 3982 (1142-13682) | 14982 (1042-26082) | **0.0088** |
| *ADHESION MOLECULES* | | | | |
| ICAM1 | 4000 | 0.00 (-33000-18000) | 0.0 (-5000-11000) | 0.5778 |
| VCAM1 | 2500 | -18000 (-116000-50000) | 12000 (-44000-52000) | 0.3836 |
| *MATRIX METALLOPROTEASES* | | | | |
| MMP3 | 51 | 300 (-3000-900) | 200 (-1000-1100) | 0.6999 |
| MMP9 | 107 000 | 59000 (0-154000) | 26000 (0-69000) | 0.0660 |

| | | | | |
|---|---|---|---|---|
| *COAGULATION FACTORS* | | | | |
| **C3** | $2.5x10^6$ | 0.00 ($-1.4x10^8$-$1.5x10^8$) | $6x10^7$ ($-8x10^7$-$1.7x10^8$) | **0.0486** |
| F7 | 28000 | -20000 (-129000-23000) | -8000 (-37000-19000) | 0.3837 |
| FGA | $1.1x10^7$ | 0.0 ($-3x10^8$-$3x10^8$) | $1x10^8$ ($-1x10^8$-$5x10^8$) | 0.0325 |
| VWF | $2.5x10^7$ | 0.0 ($-6x10^6$-$1.2x10^7$) | 0.0 ($-1.3x10^7$-$1.1x10^7$) | 0.5491 |
| *SERUM PROTEINS* | | | | |
| B2M | 5000 | 0.0 (-330000-510000) | 80000 (-70000-240000) | 0.4667 |
| FTL | 860 | 1000 (-2700-27000) | 13000 (-1000-37000) | 0.6800 |
| **HP** | $5.3x10^6$ | $4x10^7$ ($-1.8x10^8$-$7.1x10^8$) | 0.0 ($-1.6x10^9$-$0.8x10^9$) | **0.0083** |
| *TRADITIONAL MARKERS* | | | | |
| **CRP** | 48000 | -60000 ($-4x10^6$-(-10000)) | 0.0 ($-1.6x10^6$-800000) | **0.0083** |
| *OTHERS* | | | | |
| **GC** | $1.6x10^6$ | $-1.4x10^7$ ($-7.1x10^7$-$8x10^6$) | $1x10^6$ ($-5x10^7$-$1.8x10^7$) | **0.0191** |
| **SERPINA1** | $2.6x10^7$ | $-0.1x10^9$ ($-3.5x10^8$-$1x10^7$) | $7x10^7$ ($-2.4x10^8$-$1.6x10^8$) | **0.0487** |
| IL1RN | 302 | 1918 (0-3167) | 2073 (381-3448) | 0.4937 |
| KITL | 136 | 93 (56-408) | 226 (26-366) | 0.0605 |
| TIMP1 | 640 | 15000 (-6000-95000) | 21000 (-12000-37000) | 0.9886 |
| TNFRSF1B | 420 | 4400 (0-11100) | 6000 (500-9900) | 0.1151 |
