# Supplementary figures and images for: Altered Immune Activation and IL-23 Signaling in Response to Candida albicans in Autoimmune Polyendocrine Syndrome Type 1
Source: Front Immunol. 2017 Sep 1;8:1074. doi: 10.3389/fimmu.2017.01074 (PMC5585148; doi:10.3389/fimmu.2017.01074)

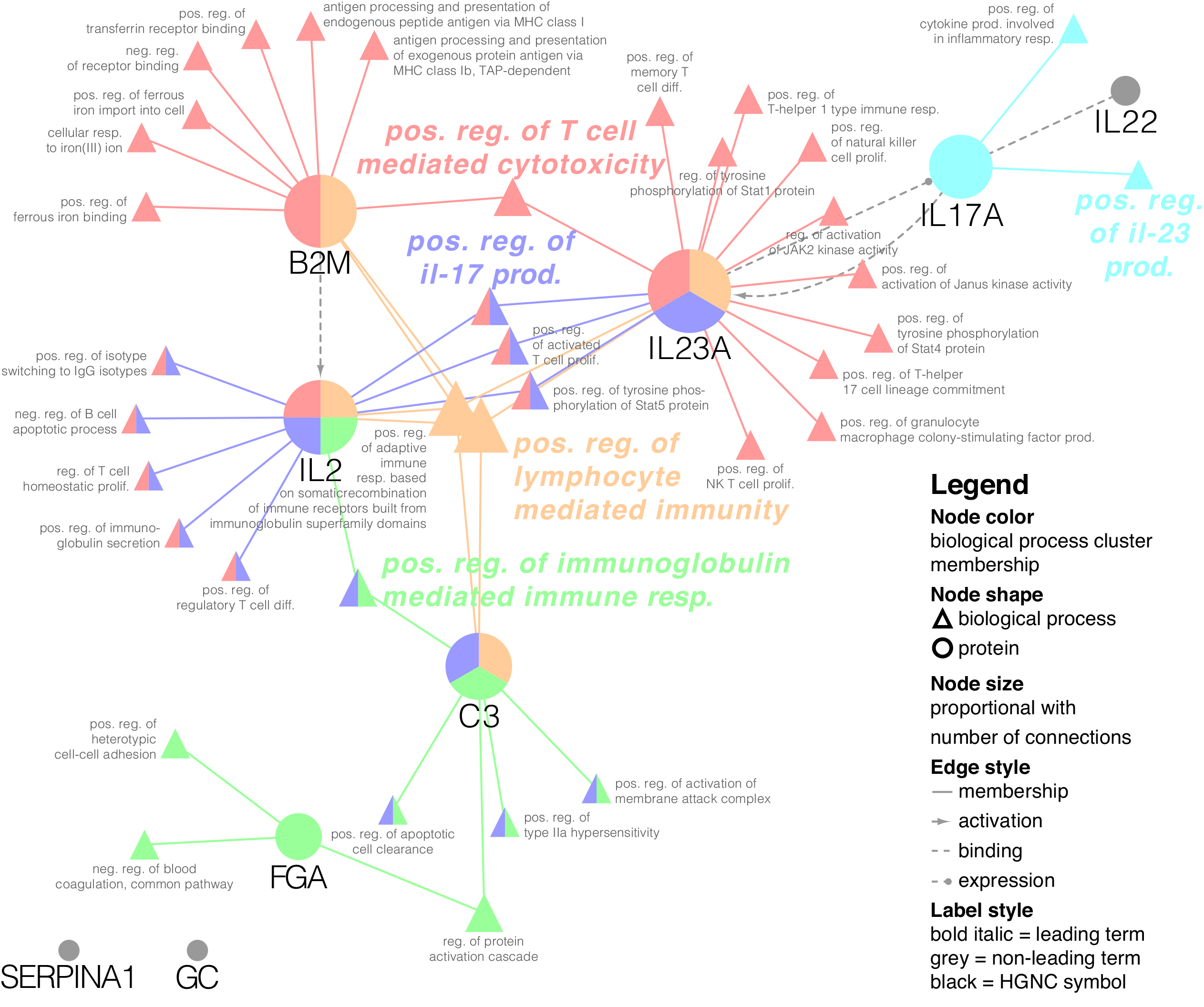

Supplement: Figure S1 — Functional annotation of the protein profile characterizing the altered immune response to C. albicans in APS-1 patients versus healthy individuals. This protein:gene-ontology-(GO) term network identified four clusters of biological processes, each with its leading term, that most efficiently interconnect the nine proteins here relevant. Any regulatory relationships between the proteins, as per CluePedia v10.0 are displayed as well. [file image_1.png]
