# Supplementary material for: Altered Immune Activation and IL-23 Signaling in Response to Candida albicans in Autoimmune Polyendocrine Syndrome Type 1
Source: Front Immunol. 2017 Sep 1;8:1074. doi: 10.3389/fimmu.2017.01074 (PMC5585148; doi:10.3389/fimmu.2017.01074)

SUPPLEMENTARY FIGURE 2. Gating strategy for flow cytometric analyses

The figure gives an overview of the IL-23 response found in monocytes in patients versus controls. **A**. Monocyte-gating according to the SSC/FSC. **B.** Gating strategy for CD14+ monocytes within the monocyte-gate from A. **C**. A representative illustration of the lack of IL-23 response in monocytes from an APS-1 patient when stimulated with curdlan. **D**. A typically IL-23 response seen in monocytes in a healthy control after stimulation with curdlan.

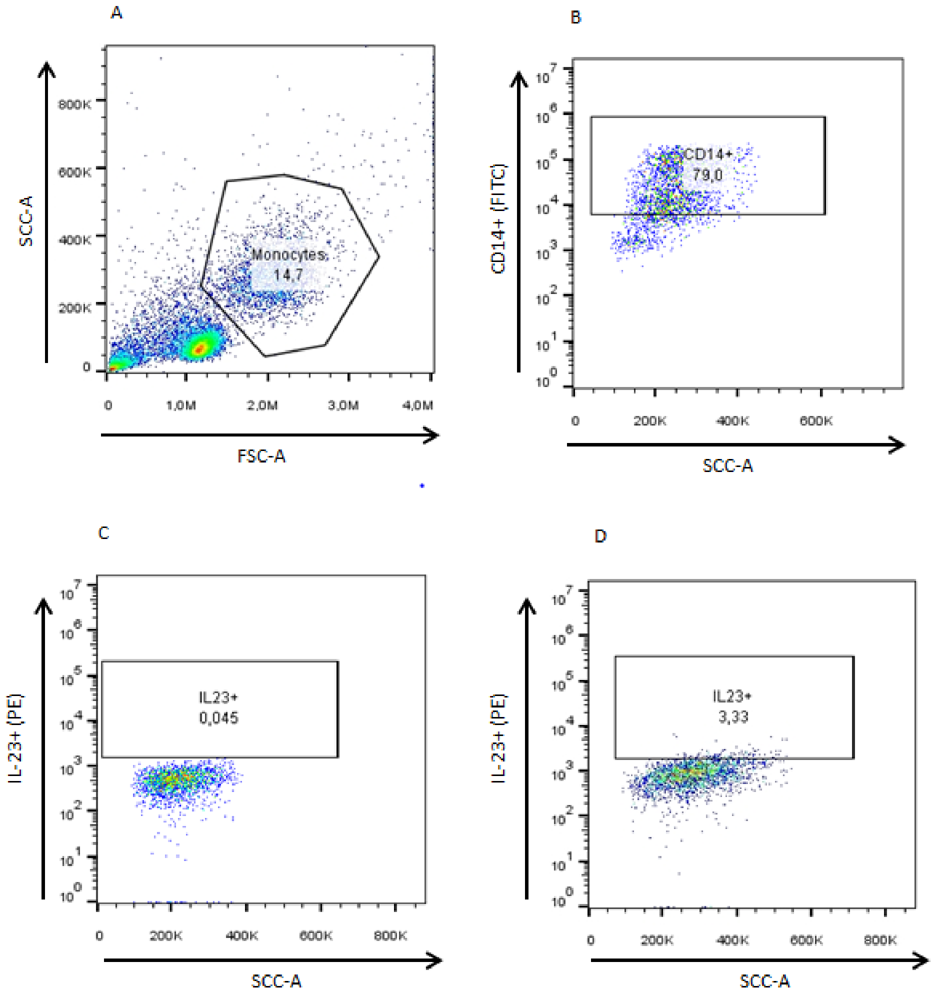

Supplement: Figure S2 — Gating strategy for flow cytometric analyses. [file image_2.pdf]
